# Myocardial Injury Portends a Higher Risk of Mortality and Long-Term Cardiovascular Sequelae after Hospital Discharge in COVID-19 Survivors

**DOI:** 10.3390/jcm11195964

**Published:** 2022-10-10

**Authors:** Riccardo Rinaldi, Mattia Basile, Carmine Salzillo, Domenico Luca Grieco, Andrea Caffè, Carlotta Masciocchi, Livia Lilli, Andrea Damiani, Giulia La Vecchia, Giulia Iannaccone, Alice Bonanni, Gennaro De Pascale, Rita Murri, Massimo Fantoni, Giovanna Liuzzo, Tommaso Sanna, Massimo Massetti, Antonio Gasbarrini, Vincenzo Valentini, Massimo Antonelli, Filippo Crea, Rocco Antonio Montone, on behalf of the Gemelli against COVID Group

**Affiliations:** 1 Department of Cardiovascular and Pulmonary Sciences, Catholic University of the Sacred Heart, 00168 Rome, Italy; 2 Department of Emergency, Intensive Care Medicine and Anaesthesia, Fondazione Policlinico Universitario Agostino Gemelli IRCCS, 00168 Rome, Italy; 3 Istituto di Anestesiologia e Rianimazione, Università Cattolica del Sacro Cuore, 00168 Rome, Italy; 4 UOC Radioterapia Oncologica, Dipartimento di Diagnostica per Immagini, Radioterapia Oncologica ed Ematologia, Fondazione Policlinico Universitario Agostino Gemelli IRCCS, 00168 Rome, Italy; 5 Dipartimento di Scienze di Laboratorio e Infettivologiche, Fondazione Policlinico Universitario Agostino Gemelli IRCCS, 00168 Rome, Italy; 6 Clinic of Infectious Diseases, Catholic University of the Sacred Heart, 00168 Rome, Italy; 7 Department of Cardiovascular Sciences, Fondazione Policlinico Universitario Agostino Gemelli IRCCS, 00168 Rome, Italy; 8 Dipartimento di Medicina e Chirurgia Traslazionale, Università Cattolica del Sacro Cuore, 00168 Rome, Italy; 9 Dipartimento di Scienze Radiologiche ed Ematologiche, Università Cattolica del Sacro Cuore, 00168 Rome, Italy

**Keywords:** myocardial injury, COVID-19, long COVID, prognosis

## Abstract

*Background:* Cardiovascular sequelae after COVID-19 are frequent. However, the predictors for their occurrence are still unknown. In this study, we aimed to assess whether myocardial injury during COVID-19 hospitalization is associated to CV sequelae and death after hospital discharge. *Methods:* In this prospective observational study, consecutive patients who were admitted for COVID-19 in a metropolitan COVID-19 hub in Italy, between March 2021 and January 2022, with a ≥ 1 assessment of high sensitivity cardiac troponin I (hs-cTnI) were included in the study, if they were alive at hospital discharge. Myocardial injury was defined as elevation hs-cTnI > 99th percentile of the upper reference limit. The incidence of all-cause mortality and major adverse cardiovascular and cerebrovascular events (MACCE, including cardiovascular death, admission for acute or chronic coronary syndrome, hospitalization for heart failure, and stroke/transient ischemic attack) at follow-up were the primary outcomes. Arrhythmias, inflammatory heart diseases, and/or thrombotic disorders were analyzed as well. *Results:* Among the 701 COVID-19 survivors (mean age 66.4 ± 14.4 years, 40.2% female), myocardial injury occurred in 75 (10.7%) patients. At a median follow-up of 270 days (IQR 165, 380), all-cause mortality (21.3% vs. 6.1%, *p* < 0.001), MACCE (25.3% vs. 4.5%, *p* < 0.001), arrhythmias (9.3% vs. 5.0%, *p* = 0.034), and inflammatory heart disease (8.0% vs. 1.1%, *p* < 0.001) were more frequent in patients with myocardial injury compared to those without. At multivariate analysis, myocardial injury (HR 1.95 [95% CI:1.05–3.61]), age (HR 1.09 [95% CI:1.06–1.12]), and chronic kidney disease (HR 2.63 [95% CI:1.33–5.21]) were independent predictors of death. Myocardial injury (HR 3.92 [95% CI:2.07–7.42]), age (HR 1.05 [95% CI:1.02–1.08]), and diabetes (HR 2.35 [95% CI:1.25–4.43]) were independent predictors of MACCE. *Conclusion:* In COVID-19 survivors, myocardial injury during the hospital stay portends a higher risk of mortality and cardiovascular sequelae and could be considered for the risk stratification of COVID-19 sequelae in patients who are successfully discharged.

## 1. Introduction

Coronavirus disease-2019 (COVID-19) is a global pandemic that has been caused by the novel severe acute respiratory syndrome coronavirus 2 (SARS-CoV-2), which continues to cause considerable morbidity and mortality worldwide [1]. Even though the clinical manifestations of COVID-19 mainly involve the respiratory tract, a significant proportion of patients may demonstrate biomarker evidence of myocardial injury (defined as increased cardiac troponin levels). Multiple mechanisms have been proposed to explain the occurrence of myocardial injury, including indirect cytokine-mediated damage, oxygen supply–demand imbalance, ischemic injury from microvascular thrombi formation, direct viral invasion of the myocardium, endothelial cells infection and endotheliitis, autoimmunity triggered by the host’s response to the virus, and acute coronary syndrome from the acute inflammation-triggered destabilization of atherosclerotic plaques [2,3,4,5,6,7]. Notably, the occurrence of myocardial injury in the acute phase of COVID-19 infection has been associated with worse in-hospital clinical outcomes [8,9,10,11,12]. Similar to the post-acute viral syndromes described in survivors of other virulent coronavirus epidemics, there are increasing reports of persistent and prolonged effects after acute COVID-19, involving, in particular, the cardiovascular (CV) system [13,14,15,16,17]. However, data regarding the long-term CV sequelae of COVID-19 are still scarce and, importantly, the long-term prognostic implications of myocardial injury, occurring in the acute phase of COVID-19, are still largely unknown. Indeed, only a few studies to date have investigated the prognostic impact of myocardial injury in COVID-19 survivors, and these studies have involved small patient populations, with a short duration of follow-up and/or a narrow selection of CV outcomes (i.e., mainly reporting its association with an increased mortality after hospital discharge) [18,19,20]. Therefore, the aim of this study was to assess the incidence and predictors of a wide range of CV sequelae at long-term follow-up, in a large cohort of hospitalized COVID-19 survivors. The aim of this was, in particular, to evaluate the role of myocardial injury, occurring during the index COVID-19 hospitalization, in modifying the risk of future CV events.

## 2. Materials and Methods

### 2.1. Study Population

The study population was obtained from the COVID-19 Data Mart, a prospectively enrolling, real-time archive of structured and unstructured data, refreshed on a daily basis, including all patients admitted for COVID-19 at Fondazione Policlinico A. Gemelli IRCCS Hospital—a main hub for COVID-19 patients in Rome, Italy [21]. In particular, the study cohort included all patients admitted from March 2021 to January 2022, who were ≥18-years-old, had a positive nasopharyngeal swab for SARS-CoV-2 within the first 48 h of admission, who had at least one assessment of high sensitivity cardiac troponin I (hs-cTnI) within the first 30 days from admission, who were discharged alive from the index hospitalization, and who had ≥1 clinical follow-up visit (Figure S1). The diagnosis of SARS-CoV-2 infection was considered positive when the reverse transcription polymerase chain reaction (PCR) of the SARS-CoV-2 assay was detected from a nasopharyngeal swab. Myocardial injury was defined as the elevation of high sensitivity cardiac troponin I (hs-cTnI) levels > 99th percentile of the upper reference limit (>56 ng/L for a normal population).

### 2.2. Data Collection

Variables collected from the index hospitalization included demographics, comorbidities, vital signs, laboratory measurements, vaccination status for COVID-19, and clinical outcome during the index hospitalization (see Appendix A for further details). All data were extracted from the electronic medical records (EMR) of all patients. To obtain structural information from unstructured texts (such as clinical diaries, radiology reports, etc.), natural language processing (NLP) algorithms were applied, based on text mining procedures, such as the following: sentence/word tokenization; rule-based approach, supported by annotations defined by the clinical subject matter experts (SMEs); and using semantic/syntactic corrections where necessary [22].

### 2.3. Study Endpoints

All patients received a clinical follow-up by telephonic interview and/or clinical visit every 3 months from hospital discharge, for up to 18 months or up to the first occurrence of a major adverse CV and cerebrovascular event (MACCE). The primary endpoints were the incidence of the following: (1) all-cause mortality at follow-up and (2) MACCE at follow-up. MACCE were defined as the composite of CV death, admission for ischemic heart disease (IHD) (including both acute coronary syndrome and chronic coronary syndrome), stroke/transient ischemic attack (TIA), and hospitalization for heart failure (HF). In addition, we also recorded the incidence of arrhythmias, inflammatory heart disease, and thrombotic disorders at follow-up. Arrhythmias were defined as the composite of new-onset atrial fibrillation (AF) and/or ventricular tachycardia (sustained or non-sustained). Inflammatory heart diseases were defined as the composite of pericarditis and/or myocarditis. Thrombotic disorders were defined as the composite of pulmonary embolism, deep vein thrombosis and/or superficial vein thrombosis (see Appendix A for further details).

### 2.4. Statistical Analysis

Continuous variables were expressed as mean ± standard deviation (SD), or as median and interquartile range, respectively, in the case they were normally or not normally distributed, and data were compared using Student’s t-test or Mann–Whitney U-test, as appropriate. Quantitative data distribution was assessed using the Shapiro–Wilk test. Categorical data were reported as absolute and relative percentage frequencies and, between groups, differences were evaluated using the χ^2^ test or Fisher exact test, as appropriate. A value of *p* < 0.05 was considered significant. Univariable Cox regression analysis was applied to assess the relation of individual variables with all-cause mortality. Cox regression was then applied to identify variables independently associated with all-cause mortality. To this end, only variables showing *p* ≤ 0.05 at univariable analysis were included in the multivariable model. Likewise, univariable Cox regression analysis was applied to assess the relation of individual variables with MACCE. Cox regression was then applied to identify the variables independently associated with MACCE. To this end, only variables showing *p* ≤ 0.05 at univariable analysis were included in the multivariable model. A two-tailed analysis was performed and a *p*-value < 0.05 was considered as statistically significant. All analyses were performed using SPSS version 21 (SPSS Inc., Chicago, IL, USA).

## 3. Results

### 3.1. Baseline Characteristics of Study Population

A total of 701 hospitalized COVID-19 survivors (mean age 66.4 ± 14.4 years, 282 [40.2%] female) were included in the study. Myocardial injury occurred in 75 (10.7%) patients during their index hospitalization for COVID-19. The patients with myocardial injury, compared to those without, were older (73.5 ± 14.6 vs. 65.5 ± 14.2 years, *p* < 0.001); had a higher prevalence of chronic kidney disease (CKD) (14 [18.7%] vs. 40 [6.4%], *p* < 0.001); type 2 diabetes mellitus (T2DM) (20 [26.7%] vs. 102 [16.3%], *p* = 0.025); hypertension (50 [66.7%] vs. 289 [46.2%], *p* = 0.001); a history of HF (13 [17.3%] vs. 30 [4.8%], *p* < 0.001); and had paroxysmal/persistent AF (14 [18.7%] vs. 49 [7.8%], *p* = 0.002). Moreover, patients with myocardial injury, compared to those without, had lower levels of hemoglobin (12.4 ± 2.2 vs. 13.7 ± 1.9 g/dL, *p* < 0.001); total protein (64.3 ± 7.0 vs. 66.4 ± 6.2 g/L, *p* = 0.017); and antithrombin (91.0 ± 14.7 vs. 102.0 ± 17.0%, *p* < 0.001). In addition, they also had higher white blood cell counts (WBC) (9.9 ± 5.7 vs. 7.8 ± 6.4 × 10^9^/L, *p* = 0.006); hs-cTnI (2606.1 ± 12,141.6 vs. 13.3 ± 12.1 ng/L, *p* < 0.001); D-dimer (3869.7 ± 5686.6 vs. 1794.5 ± 4086.7 ng/mL, *p* < 0.001); N-terminal prohormone of brain natriuretic peptide (NT-proBNP) (7612.3 ± 11,908.9 vs. 1212.6 ± 3775.8 pg/mL, *p* < 0.001); C-reactive protein (CRP) (86.3 ± 72.0 vs. 69.1 ± 61.4 mg/L, *p* = 0.025); and procalcitonin (PCT) (2.6 ± 10.1 vs. 0.4 ± 2.2 ng/mL, *p* < 0.001). Additionally, patients with myocardial injury, compared to those without, had a longer length of hospitalization (23.8 ± 22.0 vs. 15.3 ± 12.4 days, *p* < 0.001) and a higher need for mechanic ventilation (15 [20.0%] vs. 45 [7.2%], *p* < 0.001) and admission to the intensive care unit (ICU) (32 [42.7%] vs. 93 [14.9%], *p* < 0.001). Notably, there were no differences in the number of patients vaccinated against COVID-19, in the total doses of the COVID-19 vaccine administered, in the type of COVID-19 vaccine administered, the time from last dose of vaccine to troponin assessment, or in the time from the last dose of the vaccine to a positive PCR test for SARS-CoV-2, according to the presence or absence of myocardial injury during the index hospitalization (all *p* > 0.05). The baseline characteristics of the overall population, according to the presence or absence of myocardial injury are shown in Table 1.

### 3.2. Clinical Outcomes, According to the Presence or Absence of Myocardial Injury

**Figure 1:**
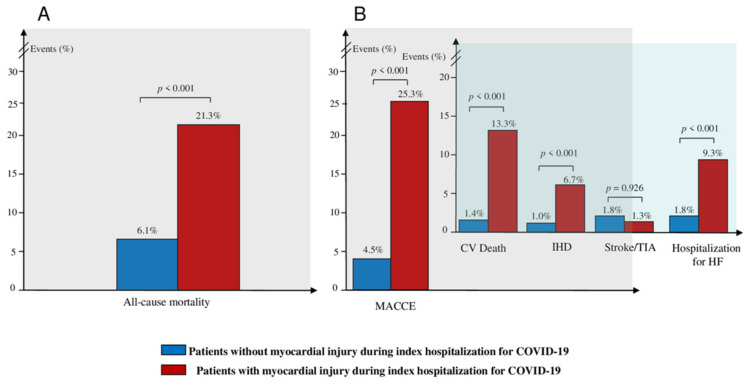
**Clinical outcome according to the presence or absence of myocardial injury during the index hospitalization:** (**A**) Incidence of composite of all-cause mortality at follow-up, according to the presence or absence of myocardial injury during the index hospitalization for COVID-19. (**B**) Incidence of composite of MACCE and individual components of MACCE at follow-up, according to the presence or absence of myocardial injury during the index hospitalization for COVID-19. *Abbreviations:* MACCE—major adverse cardiovascular and cerebrovascular event; CV—cardiovascular; IHD—ischemic heart disease; TIA—transient ischemic attack; HF—heart failure; COVID-19—Coronavirus Disease-2019.

The median follow-up time was 270 days (interquartile range [IQR] 165, 380 days) and, of note, patients with myocardial injury had a lower follow-up time compared to those without (201 [IQR 130; 357] vs. 272 [IQR 168; 381.25] days, *p* = 0.006). All-cause mortality occurred in 54 (7.7%) patients. The incidence of all-cause mortality was higher among the patients with myocardial injury during the index hospitalization for COVID-19, compared to those without (16 [21.3%] vs. 38 [6.1%], *p* < 0.001) (Figure 1A). MACCE occurred in 47 (6.7%) patients. The incidence of MACCE was higher among the patients with myocardial injury during the index hospitalization for COVID-19, compared to those without (19 [25.3%] vs. 28 [4.5%], *p* < 0.001), with a higher rate of CV death (10 [13.3%] vs. 9 [1.4%], *p* < 0.001), IHD (5 [6.7%] vs. 6 [1.0%], *p* < 0.001), and hospitalization for HF (7 [9.3%] vs. 11 [1.8%], *p* < 0.001), but without differences in the incidence of stroke/TIA (1 [1.3%] vs. 11 [1.8%], *p* = 0.926) (Figure 1B). Additionally, arrhythmias occurred in 38 (5.4%) patients, with a higher incidence among patients with myocardial injury during the index hospitalization for COVID-19 compared to those without (7 [9.3%] vs. 31 [5.0%], *p* = 0.034), and with a trend for a higher incidence of new onset AF (6 [8.0%] vs. 27 [4.3%], *p* = 0.072). However, ventricular tachycardia did not differ between the two groups (1 [1.3%] vs. 4 [0.6%], *p* = 0.174). Furthermore, inflammatory heart disease occurred in 13 (1.9%) patients. The incidence of inflammatory heart disease was higher among patients with myocardial injury during the index hospitalization for COVID-19 compared to those without (6 [8.0%] vs. 7 [1.1%], *p* < 0.001), with a higher rate of pericarditis (4 [5.3%] vs. 6 [1.0%], *p* = 0.001) and myocarditis (2 [2.7%] vs. 2 [0.3%], *p* = 0.004). Finally, there were no differences in the rate of thrombotic disorders, according to the presence or absence of myocardial injury, during the index hospitalization for COVID-19 (all *p* > 0.05) (Table 2).

### 3.3. Predictors of All-Cause Mortality in the Overall Population

**Figure 2:**
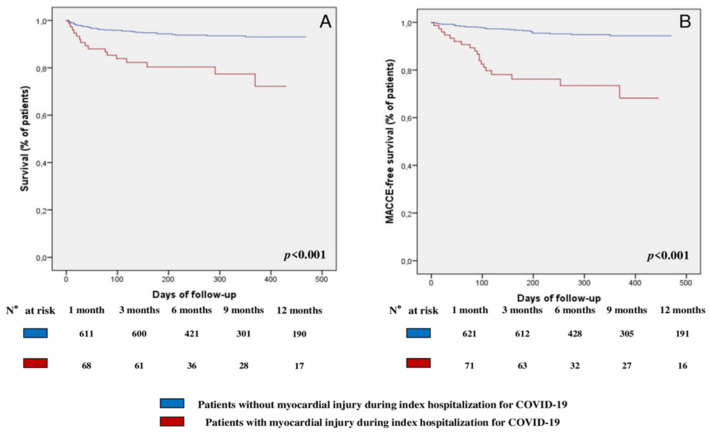
**Survival analysis:** (**A**) Survival Kaplan–Meier curve for all-cause mortality, according to the presence or absence of myocardial injury during the index hospitalization for COVID-19. (**B**) Survival Kaplan–Meier curve for MACCE, according to the presence or absence of myocardial injury during the index hospitalization for COVID-19. *Abbreviations:* MACCE—major adverse cardiovascular and cerebrovascular event; COVID-19—Coronavirus Disease-2019.

At multivariate Cox regression analysis, myocardial injury (hazard ratio [HR] 1.95, 95% confidence interval [CI] [1.05; 3.61], *p* = 0.033), older age (HR 1.09, 95% CI [1.06; 1.12], *p* < 0.001), and CKD (HR 2.63, 95% CI [1.33; 5.21], *p* = 0.005) were the only independent predictors for the occurrence of all-cause mortality in the overall population (Table 3). Comparisons of the Kaplan–Meier curves by log-rank test showed that patients with myocardial injury had a worse survival rate (*p* < 0.001, Figure 2A) compared to those without myocardial injury.

### 3.4. Predictors of MACCE in the Overall Population

At multivariate Cox regression analysis, myocardial injury (HR 3.92, 95% CI [2.07; 7.42], *p* < 0.001), older age (HR 1.05, 95% CI [1.02; 1.08], *p* < 0.001), and T2DM (HR 2.35, 95% CI [1.25; 4.43], *p* = 0.008) were the only independent predictors for the occurrence of MACCE (Table 4). Comparisons of the Kaplan–Meier curves by log-rank test showed that patients with myocardial injury had a lower MACCE-free survival (*p* < 0.001, Figure 2B) compared to those without.

## 4. Discussion

This study represents the first study that has evaluated the long-term prognostic implications of myocardial injury, occurring in the acute phase of the SARS-CoV-2 infection, on a wide range of CV sequelae in hospitalized COVID-19 survivors. The main results of our study can be summarized as follows: (1) the incidence of all-cause mortality and long-term CV sequelae in hospitalized COVID-19 survivors is considerable (7.7% for all-cause mortality, 6.7% for MACCE, 5.4% for arrhythmias, and 1.9% for inflammatory heart disease, at a median follow-up of 9 months); (2) patients who experience myocardial injury during the index hospitalization for COVID-19 have a higher incidence of all-cause mortality, MACCE, arrhythmias, and inflammatory heart disease at follow-up, compared to those who do not; (3) myocardial injury during the index hospitalization for COVID-19, older age, and CKD are independent predictors for all-cause mortality at follow-up; and 4) myocardial injury, older age, and T2DM are independent predictors for MACCE at follow-up. Previous studies have suggested that the increased risk of CV involvement in COVID-19 can extend far beyond the acute phase and can persist in the subsequent months [14,15,16,17]. Accordingly, our study demonstrated that the incidence of a wide range of CV sequelae at long-term follow-up is relevant in hospitalized COVID-19 survivors, with 6.7% of patients experiencing a MACCE. Importantly, given the large and growing number of people that are estimated to recover from COVID-19 (more than 550 million confirmed cases globally since 2019), the identification of the clinical predictors for the CV sequelae of COVID-19 would be extremely helpful for prognostic stratification. Furthermore, clinical predictors may guide the choice of therapeutic strategies in the post-acute COVID-19 phases, possibly identifying patients that may benefit from more aggressive therapies and a closer follow-up and allowing a targeted and parsimonious allocation of medical resources.

Myocardial injury is a common finding in the acute phase of the SARS-CoV-2 infection (10.7% of our population, which is consistent with previous studies, reported a prevalence ranging from 10% to 36%) and, similarly to previous studies, we found that patients with myocardial injury were older, with a higher prevalence of pre-existing comorbidities, a more pronounced inflammatory status (e.g., higher levels of WBC, D-dimer, CRP, and PCT), along with a worse in-hospital outcome (i.e., a longer length of hospitalization, a higher need for mechanic ventilation and admission to ICU) [8,9,10,11,12]. Importantly, to date, only a few studies have evaluated the prognostic role of myocardial injury after discharge in COVID-19 survivors, and they were mainly focused on the association between myocardial injury and mortality [18,19,20]. Indeed, a large study by Kini et al. showed that myocardial injury is associated with impaired survival at 6 months follow-up in 4695 hospitalized COVID-19 survivors, and that myocardial injury was an independent predictor of all-cause mortality [20]. Similarly, a study by Sanz et al., which included 256 hospitalized COVID-19 survivors, demonstrated that mortality at 1 year was significantly associated with the presence of myocardial injury during the index hospitalization [18]. Finally, Weber et al. reported that, in 377 hospitalized COVID-19 survivors, myocardial injury was significantly associated with increased 1-year mortality, and that it was an independent predictor of death at follow-up [19]. Importantly, our study expanded this notion, demonstrating, for the first time and in a large population of hospitalized COVID-19 survivors, that myocardial injury during the index hospitalization is not only associated with an increased all-cause mortality at long-term follow-up (median of 9 months after hospital discharge), but also with an increased incidence of MACCE, arrhythmias, and inflammatory heart diseases. Moreover, myocardial injury was an independent predictor of all-cause mortality (along with age, and CKD), as well as of MACCE (along with age and T2DM), even after the adjustment for potential confounding factors such as pre-existing CV conditions (i.e., CAD, HF and AF). The pathogenic mechanisms underlying these results are beyond the scope of this study. However, our findings allow us to speculate that myocardial injury, occurring in the acute phase of SARS-CoV-2 infection, may represent a marker of the underlying pathogenetic mechanisms of cardiac damage, which could be either temporally limited to the acute phase or could persist after the recovery and pose the patient an increased risk of incident CV diseases at follow-up [23,24,25]. Indeed, myocardial injury may originate directly from the viral invasion of the myocardium through the binding of SARS-CoV-2 to its host cell receptor; the angiotensin-converting enzyme 2 (ACE-2), which is widely expressed in cardiomyocytes, pericytes, and endothelial cells; or indirectly, from an enhanced immune response [5]. This latter, in particular, through the overproduction of pro-inflammatory cytokines with the consequent hyperactivation and dysregulation of the immune response (the so-called “cytokine storm”), is likely to play a central role in determining myocardial injury (as suggested by the common finding of a more pronounced inflammatory status in these patients) through several mechanisms. These mechanisms include indirect cytokine-mediated cardiac damage, a cell- or antibody-mediated autoimmune myocarditis, following the host’s response to the virus; and the presence of a prothrombotic and proinflammatory state, leading to ischemic cardiac injury, due to microvascular thrombi formation and complement-mediated coagulopathy and microangiopathy [26,27,28]. Other proposed mechanisms of cardiac damage in patients with myocardial injury include complement activation and complement-mediated coagulopathy and microangiopathy; the downregulation of the ACE-2, with the dysregulation of the renin–angiotensin–aldosterone system; autonomic dysfunction; endothelial cell infection with endotheliitis and inflammation-triggered progression; and/or the destabilization of atherosclerotic plaques [6,29,30,31,32]. Furthermore, other sources, beyond the heart. may be responsible for the release of troponin in COVID-19. Indeed, previous studies have reported that the lymphatic system has cardiac cells that express troponin C and I, as well as cardiac isoforms of tropomyosin and, therefore, the infection of the endothelial cells during COVID-19 may determine lymphatic dysfunction and troponin elevation [33,34]. However, regardless of the underlying mechanism leading to myocardial injury, the resultant cardiac damage can determine a lingering cardiac inflammatory response, with morphological and functional alterations (i.e., myocardial fibrosis and/or myocardial oedema) that persist in the post-acute phases and that could explain the increased risk of IHD, HF, dysrhythmias, inflammatory heart disease, and mortality at follow-up in these patients [31,32,35]. Supporting this hypothesis of long-lasting cardiac damage in COVID-19, mainly driven by a persistent cardiac inflammation, a recent study of cardiac magnetic resonance (CMR) in 100 patients who had recently recovered from COVID-19 (median of 71 days from COVID-19 diagnosis) showed signs of ongoing myocardial inflammation in up to 60% of the patients [36]. Moreover, a study that involved 148 hospitalized COVID-19 survivors with myocardial injury in the acute phase, who were undergoing subsequent CMR (at a median time of 68 days from hospital discharge), showed that the most prevalent pattern of myocardial injury in the post-acute phases was myocarditis-like (up to 27% of patients, and a third of these also showed evidence of ongoing myocardial inflammation), followed by ischaemia-like (22%), and dual pathology in 6% of patients [37]. Notably, this is also the first study that has evaluated the long-term prognostic implications of COVID-19 vaccines in hospitalized COVID-19 survivors and, interestingly, we found that vaccines had no role in modifying the risk of mortality and MACCE at follow-up. Such a finding is, however, not surprising, as COVID-19 vaccines certainly play a central role in the primary prevention of SARS-CoV-2 infection, but they are likely to play no role in the secondary prevention once the pathogenetic mechanisms of myocardial injury associated with SARS-CoV-2 infection are established.

### Study Limitations

Some limitations of our study should be acknowledged. First, this was a single center study. Second, cardiac troponin assessment was not systematically performed in all the COVID-19 patients referred to our center, and this could have led to selection bias. Third, the highest troponin value during the index hospitalization was used to identify the presence of myocardial injury, but further delineation of the kinetics of troponin release over time could have provided further insights. Fourth, data on the characterization of myocardial injury through cardiac imaging (especially CMR) were not available for this study, also for epidemiological reasons (e.g., restriction from infection control measures, and a lack of staff and equipment). A comprehensive diagnostic evaluation with cardiac imaging could have provided further insights into the myocardial function and the structural changes associated with the adverse CV outcomes and the increased mortality in these patients. Finally, there were limitations due to the use of EMR and NLP algorithms for data collection in such a large sample size, which was not explicitly verified by a manual chart review. However, the use of EMR enabled a timely analysis and a rapid dissemination of crucial information in a large patient cohort during a global pandemic.

## 5. Conclusions

In conclusion, our results suggest that the clinical utility and the rationale for the assessment of hs-cTnI in patients hospitalized for COVID-19 may be twofold. Indeed, previous studies demonstrated that the presence of myocardial injury in the acute phase may identify patients at higher risk of in-hospital complications and adverse in-hospital outcomes, and, therefore, those to whom the highest intensity of care should be directed. Our study supports and expands this notion, suggesting that the recognition of myocardial injury also has relevant long-term prognostic implications, representing an important predictor of mortality and CV events at follow-up in patients who survived the acute phase of COVID-19. Therefore, the assessment of hs-cTnI in the acute phase of the SARS-CoV-2 infection might be an easily available and cheap tool for the prognostic stratification of COVID-19 patients, and the choice of the best in-hospital and post-discharge management strategies. Notably, further studies are required to elucidate the pathological and molecular pathways underlying the association of myocardial injury with the adverse CV outcomes, and to identify potential pharmacological targets for the development of specific therapies that aim to mitigate the CV risk and the long-term sequelae of COVID-19. Indeed, beyond an aggressive management of the CV risk factors and a closer clinical monitoring, it is still unclear which further measures could be effectively implemented to reduce the long-term CV consequences of COVID-19 in these patients.

## Figures and Tables

**Table 1:** Baseline characteristics of the overall population, according to the presence or absence of myocardial injury during index hospitalization for COVID-19.

Characteristics	Overall Population (*n* = 701)	Patients with Myocardial Injury (*n* = 75)	Patients without Myocardial Injury (*n* = 626)	*p* Value
**Clinical characteristics**				
Age [mean ± SD]	66.4 ± 14.4	73.5 ± 14.6	65.5 ± 14.2	**<0.001**
Female sex [n, (%)]	282 (40.2)	28 (37.3)	252 (40.6)	0.588
CKD (eGFR < 60 mL/min per 1.73 m^2^) [n, (%)]	54 (7.7)	14 (18.7)	40 (6.4)	**<0.001**
History of cancer [n, (%)]	106 (15.1)	13 (17.3)	93 (14.9)	0.571
T2DM [n, (%)]	122 (17.4)	20 (26.7)	102 (16.3)	**0.025**
Hypertension [n, (%)]	339 (48.4)	50 (66.7)	289 (46.2)	**0.001**
History of HF [n, (%)]	43 (6.1)	13 (17.3)	30 (4.8)	**<0.001**
COPD [n, (%)]	87 (12.4)	14 (18.7)	73 (11.7)	0.082
Asthma [n, (%)]	29 (4.1)	4 (5.3)	25 (4.0)	0.539
Paroxysmal/persistent AF [n, (%)]	63 (9.0)	14 (18.7)	49 (7.8)	**0.002**
History of CAD [n, (%)]	91 (13.0)	15 (20.0)	76 (12.1)	0.056
DBP at admission (mmHg) [mean ± SD]	79.3 ± 12.0	77.0 ± 13.9	79.5 ± 11.8	0.134
SBP at admission (mmHg) [mean ± SD]	133.0 ± 20.9	131.6 ± 23.4	133.2 ± 20.6	0.626
Length of index hospitalization (days) [mean ± SD]	16.2 ± 14.0	23.8 ± 22.0	15.3 ± 12.4	**<0.001**
Need for mechanic ventilation [n, (%)]	60 (8.6)	15 (20.0)	45 (7.2)	**<0.001**
Need for ICU admission [n, (%)]	125 (17.8)	32 (42.7)	93 (14.9)	**<0.001**
Vaccinated (≥2 doses *) against COVID-19 [n, (%)]	241 (34.4)	32 (42.7)	209 (33.4)	0.110
Total COVID-19 vaccine doses administered [n, (%)]				0.070
Not vaccinated [n, (%)]	413 (58.9)	35 (46.7)	378 (60.4)	
1 dose [n, (%)]	47 (6.7)	8 (10.7)	39 (6.2)	
2 doses* [n, (%)]	190 (27.1)	23 (30.7)	167 (26.7)	
3 doses [n, (%)]	51 (7.3)	9 (12.0)	42 (6.7)	
Type of COVID-19 vaccine [n, (%)]				0.116
Pfizer/BioNTech Comirnaty [n, (%)]	165 (23.5)	6 (8.0)	28 (4.5)	
Vaxzevria/AstraZeneca [n, (%)]	26 (3.7)	1 (1.3)	25 (4.0)	
Spikevax/Moderna [n, (%)]	11 (1.6)	3 (4.0)	8 (1.3)	
Jcovden/Janssen [n, (%)]	5 (0.7)	0 (0.0)	5 (0.8)	
Missing data [n, (%)]	34 (4.9)	6 (8.0)	28 (4.5)	
Time from last dose of vaccine to troponin assessment (days) [mean ± SD]	127.0 ± 84.0	122.8 ± 76.1	127.6 ± 85.1	0.772
Time from last dose of vaccine to positive PCR test for SARS-CoV-2 (days) [mean ± SD]	125.3 ± 83.9	121.2 ± 76.2	125.8 ± 85.0	0.782
**Laboratory data**				
Hb (g/dL) [mean ± SD]	13.5 ± 2.0	12.4 ± 2.2	13.7 ± 1.9	**<0.001**
WBC (×10^9^/L) [mean ± SD]	8.0 ± 6.4	9.9 ± 5.7	7.8 ± 6.4	**0.006**
Serum creatinine on admission (mg/dL) [mean ± SD]	1.1 ± 1.2	1.4 ± 1.1	1.1 ± 1.2	0.068
Total protein (g/L) [mean ± SD]	66.1 ± 6.3	64.3 ± 7.0	66.4 ± 6.2	**0.017**
Antithrombin (%) [mean ± SD]	100.9 ± 17.1	91.0 ± 14.7	102.0 ± 17.0	**<0.001**
hs-cTnI at admission (ng/L) [mean ± SD]	290.7 ± 4028.3	2606.1 ± 12,141.6	13.3 ± 12.1	**<0.001**
LDH (UI/L) [mean ± SD]	391.8 ± 302.6	441.3 ± 686.6	386.0 ± 216.9	0.137
D-dimer (ng/mL) [mean ± SD]	2009.6 ± 4321.8	3869.7 ± 5686.6	1794.5 ± 4086.7	**<0.001**
Fibrinogen (mg/dL) [mean ± SD]	505.5 ± 156.9	494.2 ± 178.3	506.8 ± 154.2	0.560
NT-proBNP (pg/mL) [mean ± SD]	1902.5 ± 5635.2	7612.3 ± 11,908.9	1212.6 ± 3775.8	**<0.001**
CRP (mg/L) [mean ± SD]	71.0 ± 62.8	86.3 ± 72.0	69.1 ± 61.4	**0.025**
PCT (ng/mL) [mean ± SD]	0.7 ± 4.1	2.6 ± 10.1	0.4 ± 2.2	**<0.001**
IL-6 (ng/L) [mean ± SD]	51.7 ± 212.3	82.4 ± 194.1	48.5 ± 214.0	0.235

**Legend to table:** COVID-19—Coronavirus Disease-2019; SD—standard deviation; CKD— chronic kidney disease; eGFR—estimated glomerular filtration rate; T2DM—type 2 diabetes mellitus; HF—heart failure; COPD—chronic obstructive pulmonary disease; AF—atrial fibrillation; CAD—coronary artery disease; DBP—diastolic blood pressure; SBP—systolic blood pressure; ICU—intensive care unit; COVID-19— Coronavirus Disease 2019; PCR—polymerase chain reaction; SARS-CoV-2—severe acute respiratory syndrome coronavirus 2; Hb—hemoglobin; WBC—white blood count; hs-cTnI—high sensitivity cardiac troponin I; LDH—lactate dehydrogenase; NT-proBNP—N-terminal prohormone of brain natriuretic peptide; CRP—C-reactive protein; PCT—procalcitonin; IL-6—interleukin-6. * or equivalent (e.g., Jcovden/Janssen vaccine, see text for more details).

**Table 2:** Clinical outcomes in the overall population and according to the presence or absence of myocardial injury during the index hospitalization for COVID-19.

Characteristics	Overall Population (*n* = 701)	Presence of Myocardial Injury (*n* = 75)	Absence of Myocardial Injury (*n* = 626)	*p* Value
All-cause mortality [n, (%)]	54 (7.7)	16 (21.3)	38 (6.1)	**<0.001**
MACCE [n, (%)]	47 (6.7)	19 (25.3)	28 (4.5)	**<0.001**
CV death [n, (%)]	19 (2.7)	10 (13.3)	9 (1.4)	**<0.001**
IHD [n, (%)]	11 (1.6)	5 (6.7)	6 (1.0)	**<0.001**
Stroke/TIA [n, (%)]	12 (1.7)	1 (1.3)	11 (1.8)	0.926
Hospitalization for HF [n, (%)]	18 (2.6)	7 (9.3)	11 (1.8)	**<0.001**
Arrhythmias [n, (%)]	38 (5.4)	7 (9.3)	31 (5.0)	**0.034**
New onset AF [n, (%)]	33 (4.7)	6 (8.0)	27 (4.3)	0.072
Ventricular arrhythmias [n, (%)]	5 (0.7)	1 (1.3)	4 (0.6)	0.174
Inflammatory heart disease [n, (%)]	13 (1.9)	6 (8.0)	7 (1.1)	**<0.001**
Pericarditis [n, (%)]	10 (1.4)	4 (5.3)	6 (1.0)	**0.001**
Myocarditis [n, (%)]	4 (0.6)	2 (2.7)	2 (0.3)	**0.004**
Thrombotic disorders [n, (%)]	15 (2.1)	1 (1.3)	14 (2.2)	0.681
Pulmonary embolism [n, (%)]	6 (0.9)	0 (0.0)	6 (1.0)	0.415
Deep vein thrombosis [n, (%)]	10 (1.4)	1 (1.3)	9 (1.4)	0.997
Superficial vein thrombosis [n, (%)]	1 (0.1)	0 (0.0)	1 (0.2)	0.736
Follow-up time [days, median (IQR)]	270 [165; 380]	201 [130; 357]	272 [168; 381.25]	**0.006**

**Legend to table:** COVID-19—Coronavirus Disease-2019; MACCE—major adverse cardiovascular and cerebrovascular events; CV—cardiovascular; IHD—ischemic heart disease; TIA—transient ischemic attack; HF—heart failure; AF—atrial fibrillation; IQR—interquartile range.

**Table 3:** Predictors of all-cause death in the overall population by univariate and multivariate Cox regression analysis.

	Univariate Analysis		Multivariable Analysis	
	HR (95% CI)	*p*	HR (95% CI)	*p*
Myocardial injury	3.97 (2.21; 7.13)	**<0.001**	1.95 (1.05; 3.61)	**0.033**
Older age	1.10 (1.07; 1.13)	**<0.001**	1.09 (1.06; 1.12)	**<0.001**
CKD	4.76 (2.54; 8.91)	**<0.001**	2.63 (1.33; 5.21)	**0.005**
History of cancer	2.20 (1.20; 4.05)	**0.011**	1.58 (0.84; 2.96)	0.153
T2DM	2.40 (1.35; 4.27)	**0.003**	1.30 (0.70; 2.41)	0.400
History of HF	3.47 (1.69; 7.11)	**0.001**	1.08 (0.48; 2.39)	0.853
History of CAD	2.77 (1.53; 5.03)	**0.001**	1.06 (0.55; 2.05)	0.850
Vaccination (≥2 doses *) against COVID-19	2.46 (1.42; 4.26)	**0.001**	1.34 (0.76; 2.37)	0.317

**Legend to table:** CKD—chronic kidney disease; T2DM—type 2 diabetes mellitus; HF—heart failure; CAD—coronary artery disease; COVID-19—Coronavirus Disease 2019; HR—hazard ratio; CI—confidence interval. All variables in Table 1 have been tested to predict all-cause death, although, only variables with *p*-value < 0.05 are shown in the table. Variables that were significantly related to all-cause death have been included in the multivariate analysis. * or equivalent (e.g., Jcovden/Janssen vaccine, see text for more details).

**Table 4:** Predictors of MACCE in the overall population by univariate and multivariate Cox regression analysis.

	Univariate Analysis		Multivariable Analysis	
	HR (95% CI)	*p*	HR (95% CI)	*p*
Myocardial Injury	6.75 (3.77; 12.11)	**<0.001**	3.92 (2.07; 7.42)	**<0.001**
Older age	1.07 (1.04; 1.10)	**<0.001**	1.05 (1.02; 1.08)	**0.001**
CKD	3.49 (1.68; 7.324	**0.001**	1.09 (0.47; 2.53)	0.842
T2DM	4.04 (2.26; 7.20)	**<0.001**	2.35 (1.25; 4.43)	**0.008**
History of HF	5.57 (2.83; 10.97)	**<0.001**	1.34 (0.59; 3.04)	0.487
Paroxysmal/persistent AF	4.15 (2.19; 7.86)	**<0.001**	1.61 (0.77; 3.38)	0.206
History of CAD	3.85 (2.10; 7.04)	**<0.001**	1.66 (0.85; 3.23)	0.138

**Legend to table**: MACCE—major adverse cardiovascular and cerebrovascular events; CKD—chronic kidney disease; T2DM—type 2 diabetes mellitus; HF—heart failure; AF—atrial fibrillation; CAD—coronary artery disease; HR—hazard ratio; CI—confidence interval. All variables in Table 1 have been tested to predict MACCE, although only variables with *p*-value < 0.05 are shown in the table. Variables that were significantly related to MACCE have been included in the multivariate analysis.

## Data Availability

All data and methods supporting the findings of this study are available from the corresponding author upon reasonable request.

## Supplementary Materials

The following supporting information can be downloaded at: https://www.mdpi.com/article/10.3390/jcm11195964/s1, Figure S1: Study flowchart. *Abbreviations:* SARS-CoV-2—severe acute respiratory syndrome coronavirus 2; COVID-19—Coronavirus Disease-2019.

## Appendix A

### Appendix A.1. Data Collection

Pre-existing conditions collected were chronic kidney disease (CKD) (defined as an estimated glomerular filtration rate < 60 mL/min per 1.73 m^2^), type 2 diabetes mellitus (T2DM), hypertension, a history of heart failure (HF), chronic obstructive pulmonary disease (COPD), asthma, paroxysmal/persistent atrial fibrillation (AF), a history of coronary artery disease (CAD), and malignancy. Vital signs included diastolic blood pressure (DBP) and systolic blood pressure (SBP) at the time of admission. Laboratory parameters included hematologic variables (hemoglobin and white blood cells), creatinine, total protein, antithrombin, high sensitivity cardiac troponin I (hs-cTnI), lactate dehydrogenase (LDH), D-dimer, fibrinogen, N-terminal prohormone of brain natriuretic peptide (NT-proBNP), C-reactive protein (CRP), procalcitonin (PCT), and interleukin-6 (IL-6) at the time of admission. For hs-cTnI, if multiple measurements were available within 30 days from admission, the peak value during the hospitalization was used. Regarding the vaccination status for COVID-19, the patients were considered vaccinated two weeks after they received their second dose in a two-dose series (e.g., Pfizer/BioNTech Comirnaty, Spikevax/Moderna, or Vaxzevria/Astrazeneca vaccines), two weeks after their first dose for single-dose vaccines (e.g., Jcovden/Janssen vaccine), or two weeks after their second dose when the specific type of COVID-19 vaccine was not known. Finally, data regarding patients’ clinical outcomes during the index hospitalization included the length of hospitalization, the need for mechanic ventilation, and the need for admission to the intensive care unit (ICU).

### Appendix A.2. Clinical Outcomes and Follow-Up

Cardiovascular deaths included all deaths attributable to myocardial ischemia and/or infarction, heart failure (HF), cardiac arrest because of other or unknown causes, or a cerebrovascular accident. Ischemic heart disease (IHD) included both acute (ACS) and chronic coronary syndrome (CCS). CCS was defined as hospital admission for a stable pattern of typical chest pain at rest, on exertion, or a combination of both, and evidence of ischemia at non-invasive stress testing, without signs of myocardial infarction (MI) (defined as the detection of a rise and fall of high sensitivity cardiac troponin I [hs-cTnI] levels, with at least one value exceeding the 99th percentile of a normal reference population, and at least one of the following: symptoms of myocardial ischemia and/or new ischemic electrocardiogram [ECG] changes, namely ST-segment, T wave abnormalities and/or pathological Q waves; and/or imaging evidence of a loss of viable myocardium or new regional wall motion abnormality, in a pattern consistent with an ischemic etiology). ACS included ST-segment elevation MI (STEMI) and non-ST-segment elevation ACS (NSTE-ACS). STEMI was defined as hospital admission for acute chest pain, new persistent ST-segment elevation (more than 20 min), hs-cTnI rise and fall, and/or new regional wall motion abnormalities. NSTE-ACS was defined as hospital admission with at least two episodes of angina at rest, or one episode lasting more than 20 min during the preceding 48 h, and normal levels (unstable angina) or a rise and fall (non-ST-segment elevation MI [NSTEMI]) of hs-cTnI levels. Transient ischemic attack (TIA) was defined as an acute loss of focal cerebral or ocular function, with symptoms lasting less than 24 h and which, after adequate investigation, was presumed to be due to embolic or thrombotic vascular disease. Stroke was defined as a neurological deficit attributed to an acute focal injury of the central nervous system by a vascular cause, including cerebral infarction, intracerebral hemorrhage, and subarachnoid hemorrhage. Hospitalization for HF was defined as worsening signs and symptoms of HF (e.g., dyspnoea, decreased exercise tolerance, fatigue, worsened end-organ perfusion, symptoms of volume overload, peripheral oedema, increasing abdominal distension or ascites, pulmonary rales/crackles/crepitation, increased jugular venous pressure, hepatojugular reflux, third heart sound or gallop, and clinically significant rapid weight gain related to fluid accumulation), signifying the failure of the primary therapeutic management strategy, resulting in the escalation of therapy and requiring hospital admission. Ventricular arrhythmic events were defined as the occurrence of non-sustained (<30 **s**) and/or sustained (≥30 **s**) ventricular tachycardia (VT) (defined as three or more consecutive premature ventricular complexes), detected at any 24-**h** ECG recording that was performed after hospital discharge. The diagnosis of pericarditis, myocarditis, pulmonary embolism, deep vein thrombosis, and superficial vein thrombosis were obtained from hospital readmissions and defined using the International Classification of Diseases (ICD)-11.
