# Supplementary figures and images for: Myocardial Injury Portends a Higher Risk of Mortality and Long-Term Cardiovascular Sequelae after Hospital Discharge in COVID-19 Survivors
Source: J Clin Med. 2022 Oct 10;11(19):5964. doi: 10.3390/jcm11195964 (PMC9573406; doi:10.3390/jcm11195964)

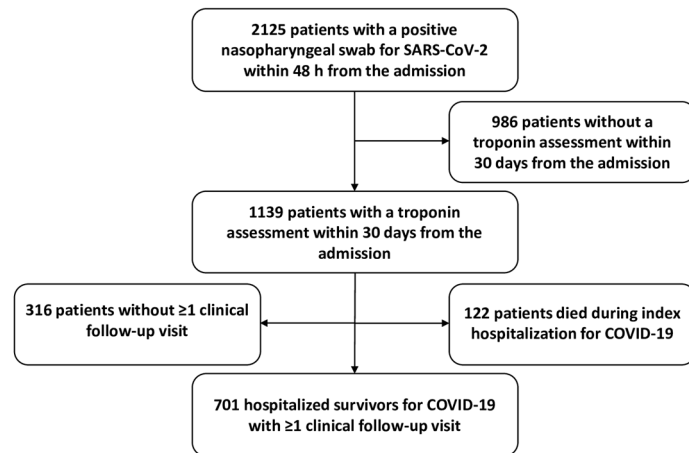

Supplement: Supplementary file 1 [file jcm-11-05964-s001.zip › jcm-1908880-supplementary.pdf]
